# Emerging trends and thematic evolution of immunotherapy for glioma based on the top 100 cited articles

**DOI:** 10.3389/fonc.2023.1307924

**Published:** 2024-01-12

**Authors:** Yan Zhou, Min Liu, Xing Huang, Zhen Liu, Yun Sun, Minjie Wang, Tao Huang, Xianke Wang, Long Chen, Xiaobing Jiang

**Affiliations:** ^1^ Department of Neurosurgery, Union Hospital, Tongji Medical College, Huazhong University of Science and Technology, Wuhan, China; ^2^ NHC Key Laboratory of Hormones and Development, Tianjin Key Laboratory of Metabolic Diseases, Chu Hsien-I Memorial Hospital and Tianjin Institute of Endocrinology, Tianjin Medical University, Tianjin, China

**Keywords:** glioma, immunotherapy, bibliometric analysis, VOSviewer, CiteSpace

## Abstract

**Purpose:**

This study aims to depict the scientific advancements in immunotherapy for glioma by analyzing the top 100 most frequently cited articles over the past 20 years.

**Methods:**

The top 100 most influential papers in immunotherapy for glioma were identified from the Web of Science Core Collection. Citations, countries/regions, institutions, journals, authorships, keywords, and references were extracted and analyzed by CiteSpace, VOSviewer, R software, and an online bibliometric platform.

**Results:**

The United States possessed a robust global presence, leading in terms of publications and maintaining strong collaborative ties with numerous countries. The institution that made the greatest contributions was Duke University, with 16 papers. Heimberger AB, Sampson JH, and Reardon DA secured the top three positions with 15, 12, and 11 papers, respectively. “Macrophage ontogeny,” “microglia,” “polarization,” “mass cytometry,” “tumor mutation burden,” “sensitivity,” “msh6,” “pd-1 blockade,” and “dna repair” were the recent hot keywords. “Microglia” and “polarization” as the emerging research directions should be given more consideration.

**Conclusions:**

This is the first bibliometric analysis to identify the top 100 papers on immunotherapy for glioma. “Microglia” and “polarization” will be hot spots for future research. The clinical efficacy of glioma immunotherapy is not yet satisfactory, and there is an urgent need to search for more tumor specific antigens and targets that can assist in early diagnosis, precise treatment, prognosis, and recurrence prediction of glioma.

## 1 Introduction

In the past two decades, significant transformations have taken place in antitumor strategies for solid tumors. In the initial 10 years, the focus shifted from traditional approaches, such as DNA replication inhibition and cell differentiation targeted therapies like receptor tyrosine kinase (RTK)–targeted therapy (1–3). The subsequent decade witnessed the advent of immunotherapy, introducing a new paradigm for both hematological and solid tumors (4). Among various immunotherapies, the emergence of immune checkpoint inhibitors (ICIs) such as anti–programmed cell death 1 (PD-1)/programmed cell death ligand 1 (PD-L1) and anti–cytotoxic T-lymphocyte–associated protein 4 (CTLA-4) has revolutionized the treatment options for aggressive forms of cancer, including melanoma, lung cancer, breast cancer, and renal cell carcinoma (5–9). Nevertheless, solid tumors often pose challenges to immunotherapy due to the immunosuppressive tumor microenvironment (TME) and physical barriers (10). To reshape the immunosuppressive microenvironment, researchers are developing more immunotherapeutic strategies (11, 12). Moreover, numerous clinical trials are underway to explore combinations involving ICIs (7, 9). Although ICIs have achieved notable success, their benefits are limited to a subset of patients.

Glioblastoma (GBM), the most lethal type of glioma, presents a “cold” immune microenvironment (13). To achieve better therapeutic effects, the adoption of new anticancer therapies, such as ICIs, vaccine therapies, and adaptive cell transfer therapy (ACT), is being developed and has proven to be beneficial to some patients (14–17). An increasing number of researchers are dedicated to overcoming the immune inhibitory microenvironment in GBM.

Bibliometrics seeks to comprehend the knowledge structure of a scientific field during a particular period (18–20). In the field of biomedicine, many bibliometric analyses have been conducted to gain insights into specific research areas (21–23). Nevertheless, the global bibliometric analysis on glioma immunotherapy has not yet been performed. The aim of this study is to present an overview of the whole scientific field and to offer new research directions by systematically evaluating the top 100 most influential papers on glioma immunotherapy over the last 20 years.

## 2 Materials and methods

### 2.1 Dataset selection

Science Citation Index Expanded of Clarivate Analytics’ Web of Science Core Collection (WoSCC), the most widely used database for bibliometric studies, is used as the data source (20, 24). The literatures between 2003 and 2022 regarding immunotherapy and glioma were retrieved on 2 April 2023. The main search terms were Title (TI) or Abstract (AB) or Author Keywords (AK): “immunotherapy” and “glioma.” The detailed searching query string is described in the **Supplementary File**. “Articles” was the only type of literature considered, whereas the language was not limited. After examining the abstract and literature type, top 100 most cited articles were downloaded in plain text (full record and cited references) for subsequent analyses. PRISMA 2020 framework was used to identify, filter, and select the relevant papers.

### 2.2 Data visualization and analysis

VOSviewer (version 1.6.16) (19) was used to construct visualization networks maps. VOSviewer thesaurus file was used to merge variants of terms and capitalize initials. Thresholds of items were described in the “Results” section. In VOSviewer map, nodes represent different objects, such as countries/regions, institutions, and researchers. The links between nodes showed the correlations between objects, which were quantitatively assessed by total link strength. The co-occurring timeline view of all keywords (author keywords and keywords plus) and citation burst of co-cited references were completed by CiteSpace (version 6.2.2) (18), and the setting parameters were as follows: years per slice (one year), selection criteria (top 50 of most cited or occurred items from each slice; g-index: k = 25), and pruning (pathfinder and pruning the merged network). More details were shown in the corresponding visualization map.

R language package (“bibliometrix”) was employed to visualize affiliations and authors’ production over time on R 4.2.0 software. Thematic map and thematic evolution of keywords were also visualized by bibliometrix package. An online bibliometric analysis platform (https://bibliometric.com/) was used to visualize cooperation relationships between countries/regions. The global publications and citation trends were performed by Microsoft Excel.

## 3 Results

### 3.1 Trend of publications and citations

**Figure 1:**
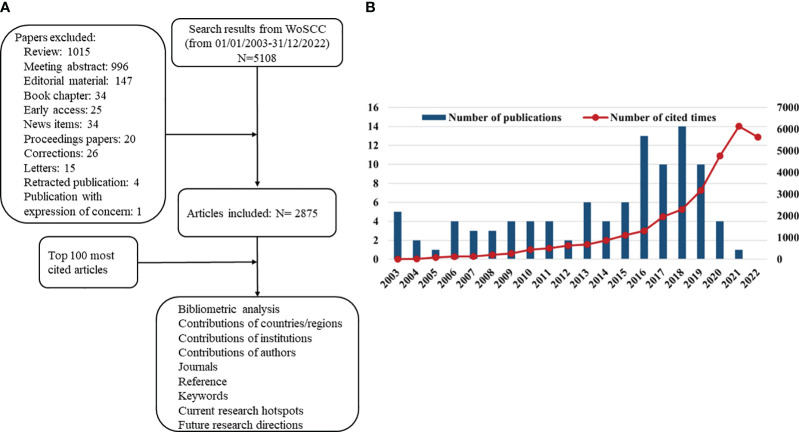
**(A)** Schematic diagram of the search process. **(B)** The annual number and total citations of top 100 most cited articles related to glioma immunotherapy published from 2003 to 2022.

From 2003 to 2022, 2,875 articles focused on glioma immunotherapy in the WoSCC (**Figure 1A**). The top 100 most cited articles were summarized in **Supplementary Table**. The citation frequency among these 100 articles ranged from 176 to 1,026. Only the article by Parsa et al. (2007) “Loss of tumor suppressor PTEN function increases B7-H1 expression and immunoresistance in glioma” received more than 1,000 citations, and half of these articles were cited more than 250 times. As for average citations per year, “Neoantigen vaccine generates intratumoral T-cell responses in phase Ib glioblastoma trial” contributed by Keskin et al. (2019) received most citations (n = 136.4).

In **Figure 1B**, high-impact articles were mainly published in 2016–2019, with the most articles published in 2018 (n = 14). The high-impact articles on glioma immunotherapy had 31,289 citations, with a steady increase in annual citations from 2003 to 2021, except for a slight decline in 2022.

### 3.2 Countries/regions and institutions analysis

**Figure 2:**
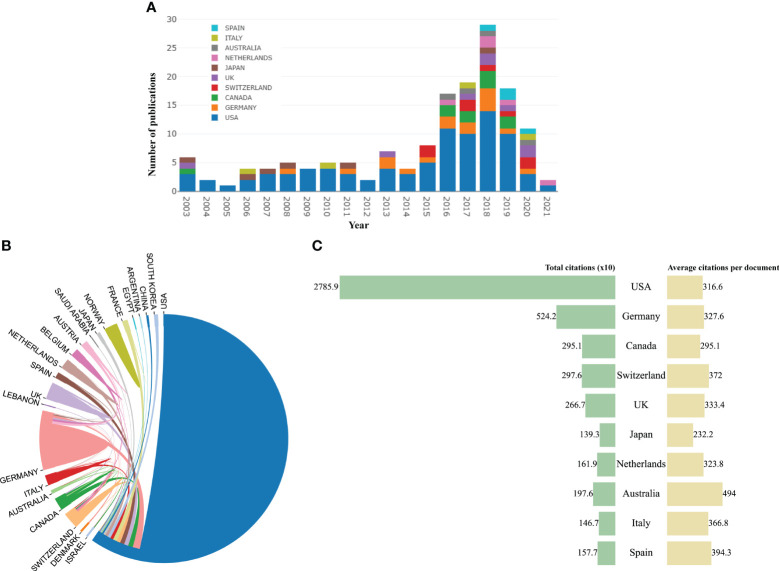
**(A)** The trend of annual number of articles from top 10 countries/regions. **(B)** The international collaborations between countries/regions. **(C)** Total citations and average citations per paper of top 10 countries/regions.

Twenty-three countries/regions in the world have published the top 100 highly cited articles. **Figure 2A** represented the annual quantity of publications of the top 10 countries in recent 20 years. The United States published the most articles (n = 88), significantly more than the second place, Germany (n = 16), and the third place, Canada (n = 10). Scientists from the USA received most citations (n = 27,859) and collaborated closely with scientists from other countries (**Figures 2B, C**). It is clear that the USA dominated this area.

**Figure 3:**
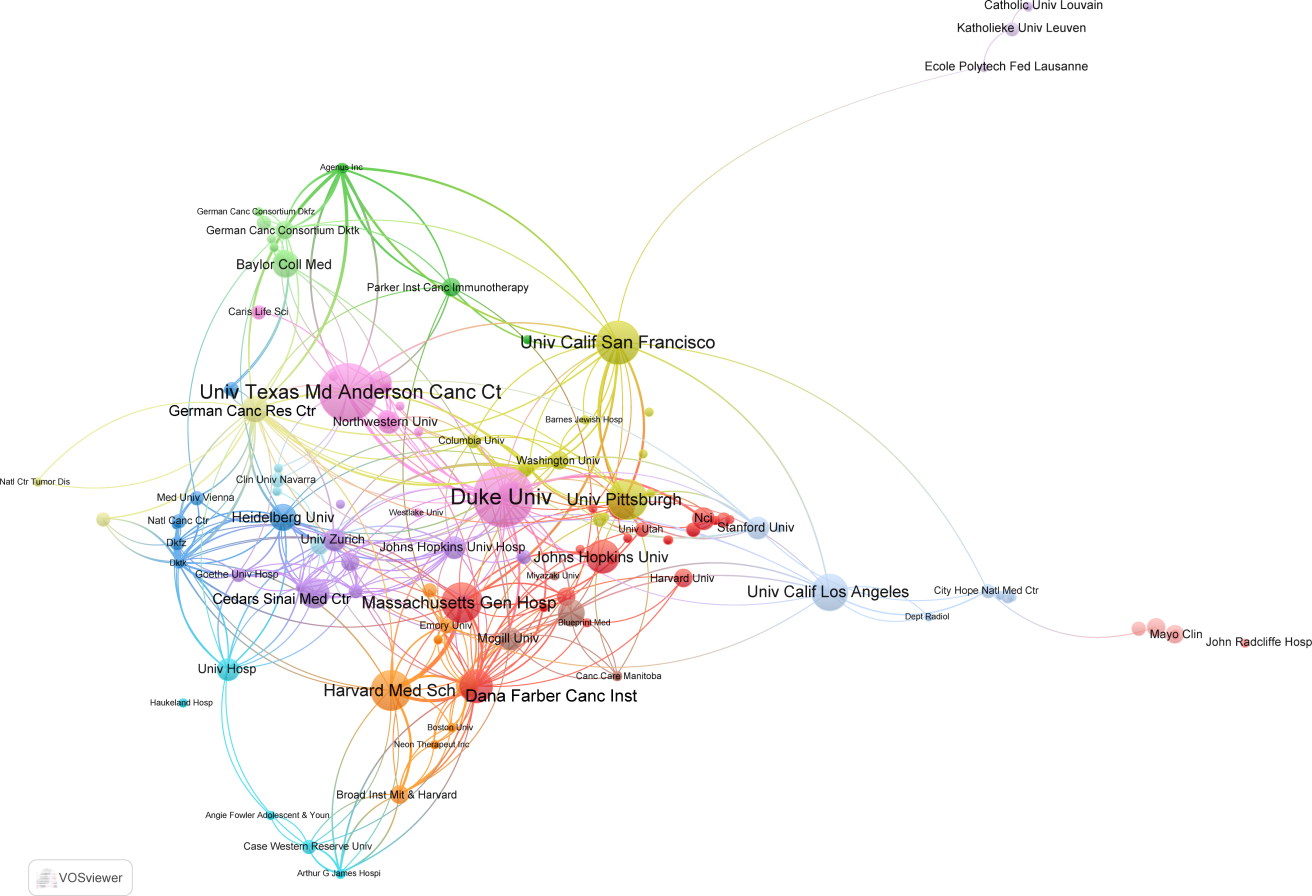
The collaboration network map of institutions performed by VOSviewer. The size of nodes represented the number of publications, whereas the thickness of lines indicated the collaboration strength.

These highly cited papers were contributed by more than 256 institutions. Unexpectedly, all the top 10 institutions were from USA according to publications (**Table 1**). Of them, Duke University ranked first with 16 top 100 articles and 4,747 citations. The second and third ranked institutions were the University of Texas MD Anderson Cancer Center and University of California, San Francisco, with 15 and 10 publications, respectively. In the collaboration network of institutions, the University of Texas MD Anderson Cancer Center, Duke University, and Dana-Farber Cancer Institute had close collaboration with other institutions (**Figure 3**).

**Table 1:** The top 10 institutions among the top 100 articles.

Rank	Institutions	Country	Articles	Citations
1	Duke University	USA	16	4,747
2	The University of Texas MD Anderson Cancer Center	USA	15	4,581
3	University of California, San Francisco	USA	10	5,012
4	Harvard Medical School	USA	9	2,783
5	Massachusetts General Hospital	USA	9	3,803
6	University of Pittsburgh	USA	9	3,113
7	University of California, Los Angeles	USA	8	3,344
8	Dana-Farber Cancer Institute	USA	7	2,594
9	Johns Hopkins University	USA	7	1,748
10	Cedars Sinai Medical Center	USA	6	1,797

### 3.3 Authors

There were 1,439 authors involved in highly cited studies on glioma immunotherapy.

**Figure 4:**
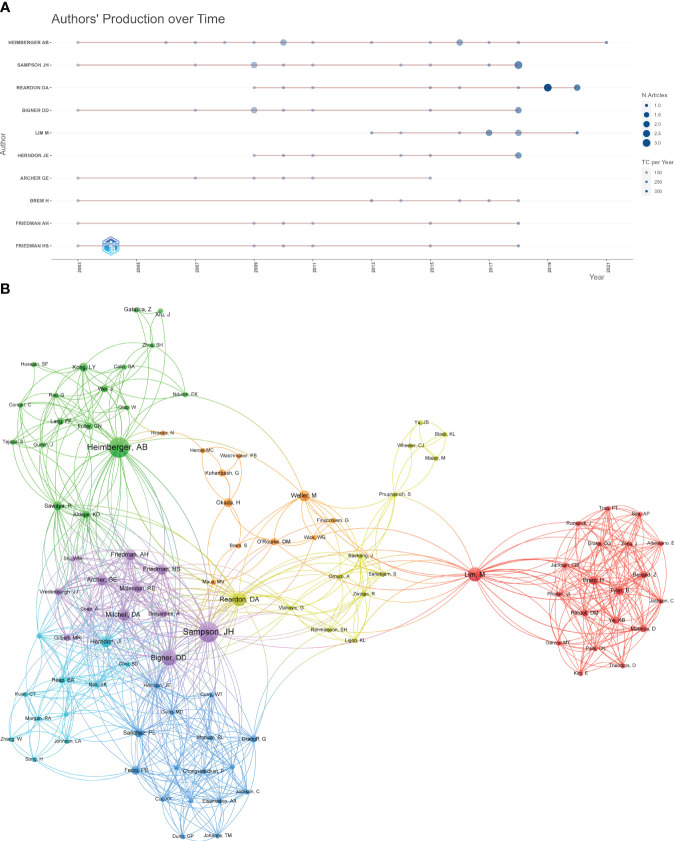
**(A)** The annual production of authors and total citation per year. **(B)** The cooperation network map of authors.

The top 10 most productive authors were listed in **Table 2**. Likewise, all of them were from the USA. Heimberger AB, Sampson JH, and Reardon DA were the top three, with 15, 12, and 11 articles, respectively. We can find that scholar Lim M (from Johns Hopkins University) received most citations per article (n = 305.1), despite having only eight articles, indicating that his results had a greater impact. The annual production of authors and total citation per year were shown in **Figure 4A**. Professor Heimberger has been publishing highly cited articles for nearly two decades, and professor Sampson’s most recent highly cited article was published in 2018.

**Table 2:** The top 10 authors in the field of glioma immunotherapy.

Rank	Author	Articles	Citations	Citations per article	Institution	Country
1	Heimberger AB	15	3,086	205.7	The University of Texas MD Anderson Cancer Center	USA
2	Sampson JH	12	3,556	296.3	Duke University	USA
3	Reardon DA	11	2,288	208.0	Dana Farber Cancer Institute	USA
4	Bigner DD	9	2,525	280.5	Duke University	USA
5	Lim M	8	2,441	305.1	Johns Hopkins University	USA
6	Herndon JE	7	1,683	240.4	Duke University	USA
7	Brem H	6	1,156	192.7	Johns Hopkins University	USA
8	Friedman AH	6	1,721	286.8	Duke University	USA
9	Archer GE	6	1,545	257.5	Duke University	USA
10	Friedman HS	6	1,721	286.8	Duke University	USA

When it comes to the co-authorship analysis, Sampson JH, Bigner DD, Reardon DA, Heimberger AB, and Lim M were the top five authors placed in the centers of the network, with the greatest total link strength (**Figure 4B**). These scholars were pioneers and highly regarded authorities in their respective fields, playing a significant role in advancing global research on immunotherapy for glioma. They possessed the necessary skills to engage in international collaboration and communication. However, additional studies are necessary to further enhance their expertise in this area.

### 3.4 Contributions of top journals

A total of 45 academic journals published top 100 highly cited papers in this research field. There were 61 articles published on the top 10 productive journals (**Table 3**). Clinical Cancer Research published the most papers [n = 17, Impact Factor (IF) 2021 = 13.801], followed by Neuro-Oncology (n = 9, IF 2021 = 13.029) and Nature Medicine (n = 7, IF 2021 = 87.244). Almost all of the top 10 journals are classified as Q1 according to the JCR 2021 criteria.

**Table 3:** The distribution of top 10 journals.

Rank	Journal	Articles	Citations	Citations per article	IF 2022	JCR 2022
1	Clinical Cancer Research	17	4,255	250.3	11.5	Q1
2	Neuro-Oncology	9	2,575	286.1	15.9	Q1
3	Nature Medicine	7	3,126	446.6	82.9	Q1
4	Cancer Research	6	1,427	237.8	11.2	Q1
5	Science Translational Medicine	6	2,237	372.8	17.1	Q1
6	Nature	5	2,158	431.6	64.8	Q1
7	Journal of Clinical Oncology	4	1,842	460.5	45.3	Q1
8	Cancer Immunology Immunotherapy	3	650	216.7	5.8	Q1/Q2
9	Cancer Immunology Research	2	421	210.5	10.1	Q1
10	Cell	2	559	279.5	64.5	Q1

IF, Impact Factor; JCR, Journal Citation Reports.

### 3.5 Citation bursts of references

**Figure 5:**
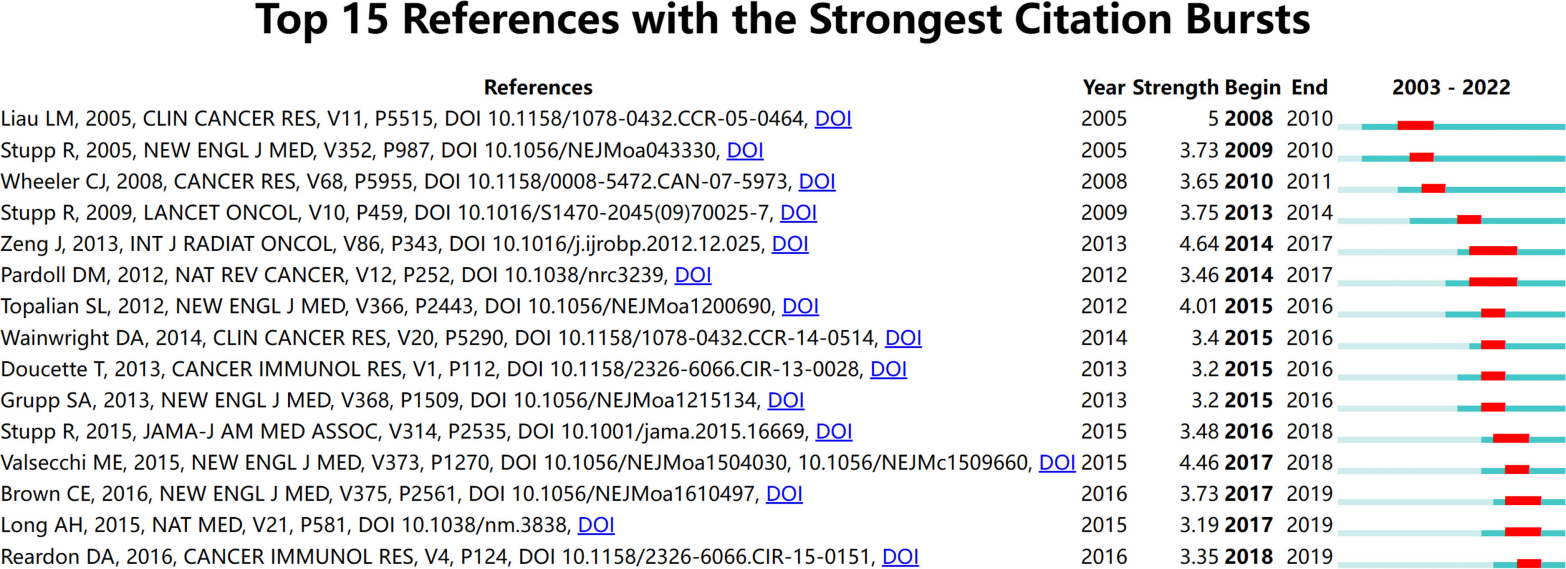
The top 15 references with the strongest citation bursts during 2003 to 2022.

Citation bursts mean that a reference was well known in this area and had been widely cited in a particular period. The red line segment indicates the active time. Fifteen references with strongest citation bursts from top 100 highly cited articles were identified by CiteSpace (**Figure 5**). The recent references with high citation were “Brown CE, 2016, NEW ENGL J MED, V375, P2561, DOI 10.1056/NEJMoa1610497” (25), “Long AH, 2015, NAT MED, V21, P581, DOI 10.1038/nm.3838” (26), and “Reardon DA, 2016, CANCER IMMUNOL RES, V4, P124, DOI 10.1158/2326-6066.CIR-15-0151” (27).

### 3.6 Keywords analysis of research hot spots

**Figure 6:**
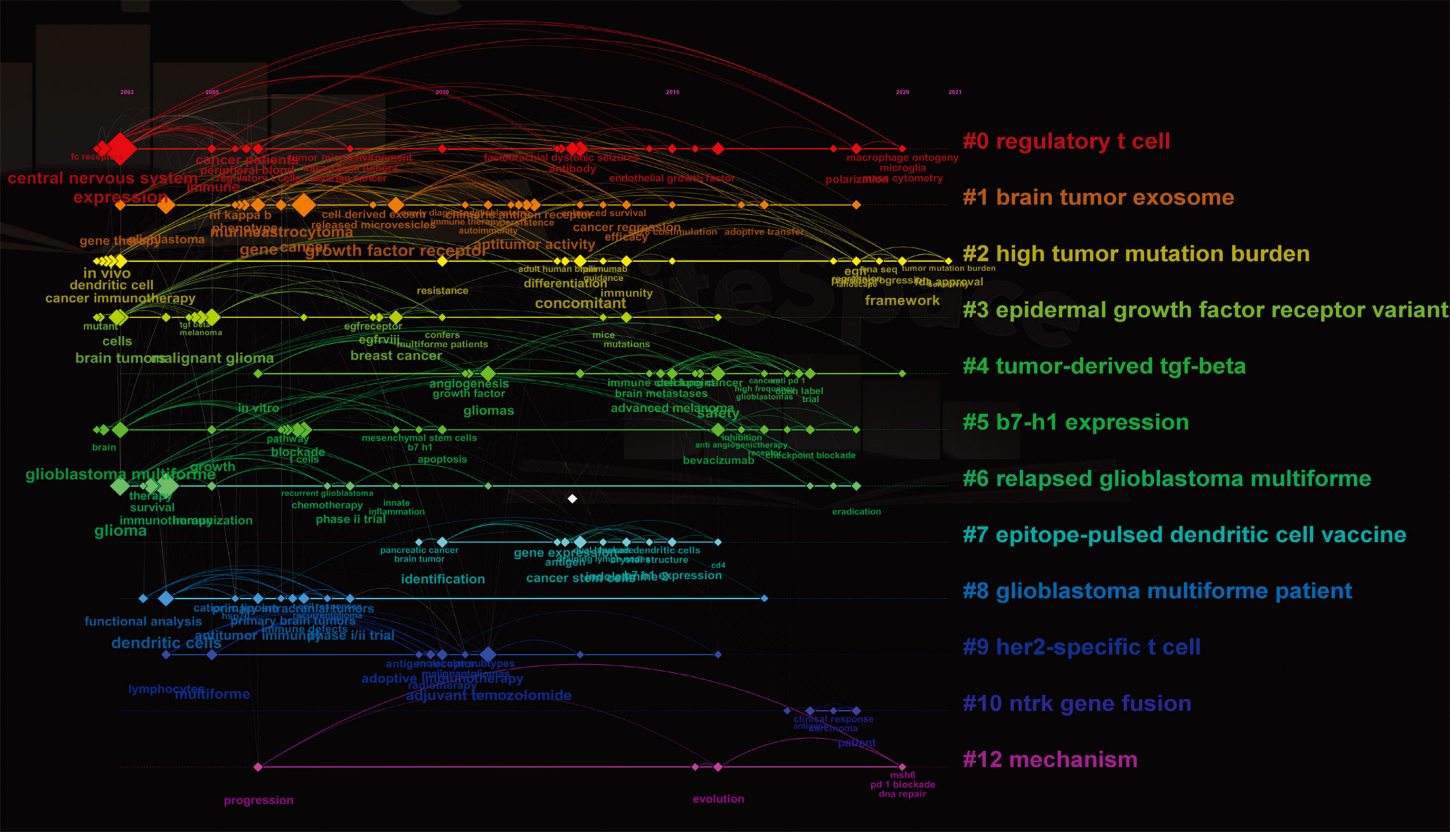
The co-occurring timeline view of all keywords (author keywords and keywords plus) visualized by CiteSpace. Thirteen labeled clusters are colored on the right. The nodes on the line represented the cited keywords.

A timeline view of co-occurring author keywords and keywords plus was performed to reflect the changes of research hot spots over time (**Figure 6**). Thirteen clusters were displayed and the nodes on the right represented recent occurring keywords, whereas the nodes on the left represented older keywords. The latest clusters on the timeline were “#0 regulatory t cell,” “#2 high tumor mutation burden,” “#4 tumor-derived tgf-beta,” and “#12 mechanism,” suggesting that more concerns are shifting to overcoming suppressive immune microenvironment.

### 3.7 Thematic evolution and thematic map

**Figure 7:**
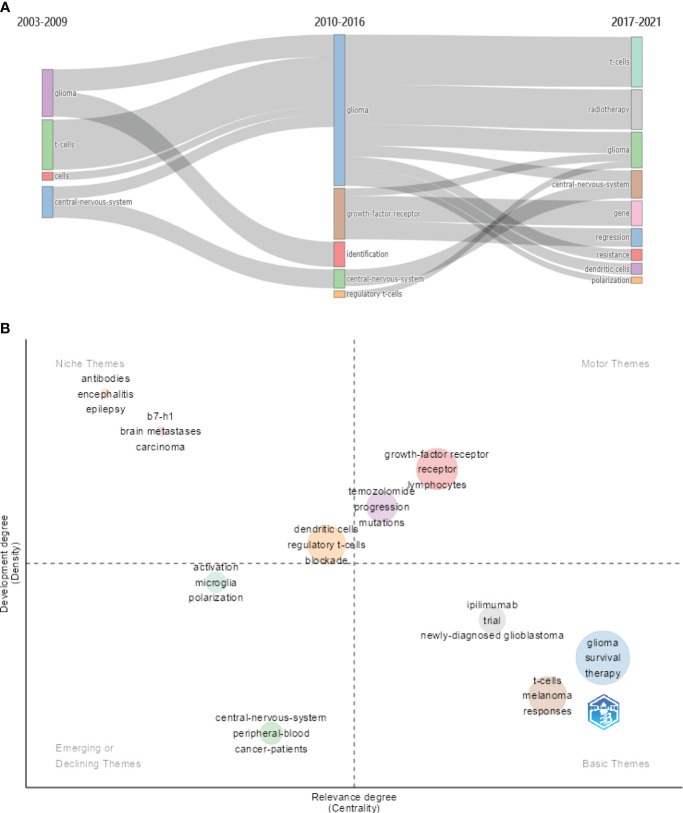
**(A)** The evolution of themes based on keywords for three subperiods. **(B)** The thematic map based on keywords.

In bibliometrics, co-occurrence analysis and cluster analysis of keywords obtained from collected literatures are usually performed in order to acquire the evolution path of research themes over time. We used R language package “bibliometrix” to visualize the evolution of themes. In **Figure 7A**, we can see that, for the first 7 years, the focus was mainly on the central nervous system and T cells, followed by the findings that some factors including “growth factor receptor” and “regulatory t cell” contributed to the highly immunosuppressive glioma microenvironment in the next 7 years. In the last 5 years, “dendritic cells” and “polarization” of immune cells have attracted more attention.

The thematic map is primarily utilized to illustrate the connections between various themes and the intensity of the connections within a theme, thereby highlighting their significance in an area and the progression of the respective themes. In **Figure 7B**, centrality (the horizontal axis) reflects the relevance of a theme in relation to other themes. Themes with higher centrality exhibit greater significance within the research area. On the other hand, density (the vertical axis) reflects the strength of the relationship among the keywords within a given theme. A theme with a higher density is indicative of a more developed or matured concept. The motor themes including “growth factor receptor,” “lymphocytes,” and “temozolomide” hold significant importance in a research domain and are well developed. Emerging or declining themes contains “microglia,” “polarization,” “activation,” “central nervous system,” and “cancer patients.”

Microglia and macrophages are common stromal cells in glioma, even accounting for 30%–50% of all cells (28). Glioma-associated microglia/macrophages (GAMs) share common histological features and differentiate into different phenotypes (mainly M1 and M2 polarization) in the presence of different induction factors. Among these highly cited articles, Saha et al. achieved significant tumor suppression in a mouse model treated with Interleukin-12 (IL-12)–expressing oncolytic viruses and ICIs, and this combination treatment promoted M1-like polarization and macrophage influx (29). The mechanisms by which glioma modulates GAM recruitment and functions are unclear. Takenaka et al. reported that kynurenine generated by GBM activated aryl hydrocarbon receptor (AHR) in GAMs to promote the recruitment of GAMs while increasing Kruppel-like factor 4 (KLF4) expression and inhibiting nuclear factor kappa-B (NF-κB) activation (30). Reprogramming these microglia/macrophages into an anti-tumor phenotype (M1) is currently a hot direction in this research area.

### 3.8 Clinical trails

Clinical trials are of utmost importance in the field of medical research as they help establish the safety and efficacy of new treatments, advance medical knowledge, and facilitate the approval of new treatments. In order to grasp the current status of clinical trials on glioma immunotherapy, we searched for clinical trials with results published on Pubmed in the last 4 years and listed them in **Table 4**. ICIs, which block the pathways that help tumor cells develop immune escape, have been tested in several clinical trials for GBM. Although some patients have shown a positive response to ICIs, the overall response rate has been low, and immune-related adverse events have been reported (31, 40). Dendritic cells (DCs) are sensitized by tumor antigens such as DNA, RNA, and tumor peptides and then activate the T-cell immune response against tumors with their powerful presentation function. A phase III clinical trial (NCT00045968) reported that some patients with GBM received autologous tumor lysate-loaded DC vaccine experienced clinically significant prolonged survival (47). Another approach is adoptive cell therapy [e.g., chimeric antigen receptor (CAR) T cell], in which immune cells are extracted from a patient, modified in the laboratory to recognize and attack cancer cells and then infused back into the patient. A phase I clinical trial (NCT04196413) covered that three of the four patients with H3K27M-mutated diffuse midline glioma showed radiographic and clinical benefits after disialoganglioside-targeted CAR T-cell administration (41). Some of early-stage clinical trials of these approaches have shown promising results, with some patients showing significant tumor shrinkage and extended survival.

**Table 4:** Clinical trials for immunotherapy in glioma in the last 4 years.

Conditions	Type of strategy	Rationale/regimen	Results	Status	Phase	Identifier
Recurrent GBM	ICIs + anti-VEGF	Pembrolizumab + bevacizumab or pembrolizumab	Pembrolizumab was ineffective as monotherapy and with bevacizumab for recurrent GBM.	Completed	II	NCT02337491 (31)
Recurrent GBM	ICIs + VEGFR inhibitor	Avelumab + axitinib or axitinib	The addition of avelumab did not provide better efficacy than axitinib alone.	Completed	II	NCT03291314 (32)
DIPG and other gliomas	Peptide vaccine + ICIs	H3.3K27M peptide vaccine + nivolumab	Patients with H3.3K27M-specific CD8^+^ immune responses showed better OS compared with non-responders.	Active	I/II	NCT02960230 (33)
Primary GBM	Adoptive cellular therapy	Autologous CMV-specific T cells	Autologous CMV-specific T cells may improve OS of patients with GBM, if offered before recurrence.	Completed	I	ACTRN12615000656538 (34)
GBM	DC vaccine	Autologous dendritic cells + autologous lymphocytes or autologous dendritic cells	Nearly one third of the patients with GBM receiving CMV-specific DC vaccines results in long-term survivors.	Active Completed	I II	NCT00639639 NCT02366728 (35)
Recurrent GBM	ICIs + anti-VEGF	Nivolumab or nivolumab + ipilimumab or bevacizumab	Nivolumab monotherapy did not improve OS compared with bevacizumab in recurrent GBM.	Active	III	NCT02017717 (15)
Pediatric CNS Tumors	CAR T cell	HER2-specific CAR T cell	The repeated intra-CNS delivery of HER2-specific CAR T cells is feasible and activate a localized immune response.	Active	I	NCT03500991 (36)
Recurrent GBM	ICIs	Ipilimumab + nivolumab	Nivolumab and ipilimumab combination following maximal safe resection of rGBM was correlated with improved OS.	Active	I	NCT03233152 (37)
GBM	Immune adjuvants + radiotherapy	GM-CSF + CAR T + TCR-T + radiotherapy	The combination of immune adjuvants and radiation may provide clinical benefit for patients with malignant glioma.	Active	I	NCT03392545 (38)
Recurrent GBM	ICIs	Pembrolizumab	Pembrolizumab showed durable antitumor activity in a subset of patients with recurrent GBM.	Completed	I	NCT02054806 (39)
Recurrent GBM or anaplastic astrocytoma	HFSRT + ICIs + anti-VEGF	Hypofractionated stereotactic irradiation (HFSRT) + pembrolizumab + bevacizumab	Adding pembrolizumab enhanced the efficacy of the combination of HFSRT and bevacizumab.	Completed	I	NCT02313272 (40)
Diffuse midline gliomas	CAR T cell + chemotherapy	GD2 CAR T cells + fludarabine + cyclophosphamide	Three of four patients showed radiographic and clinical benefit after GD2-CAR T cells administration.	Active	I	NCT04196413 (41)
Recurrent GBM	CAR T cells	Off-the-shelf, steroid-resistant, IL13Rα2-specific CAR T cells + IL-2	Four of six patients with GBM treated with CAR T cells showed the transient anti-tumor effects.	Completed	I	NCT01082926 (42)
WHO grade II glioma	Vaccine	Vaccination with tumor-cell lysate	The glioma lysate vaccines induced vaccine-reactive CD8^+^ T cells to migrate into the TME.	Active	I	NCT02549833 (43)
Recurrent and newly diagnosed GBM	DC vaccine	DC vaccine + TMZ + radiotherapy or DC vaccine + Bevacizumab	A subset of patients treated with DC vaccine showed a cytotoxic T-cell response.	Completed	I	NCT02010606 (44)
GBM	IL-12 + ICIs	IL-12 + nivolumab + veledimex	The safety of the combination of IL-12 + nivolumab was established.	Completed	I	NCT03636477 (45)
Newly diagnosed GBM	DC vaccine	DC vaccine targeting tumor-initiating cell	Median PFS was longer than historical benchmarks, although median OS was not improved.	Active	II	NCT03400917 (46)
Newly diagnosed and recurrent GBM	DC vaccine	Autologous tumor lysate-loaded dendritic cell vaccine	Adding autologous tumor lysate-loaded dendritic cell vaccine resulted in clinically significant extension of survival for patients with GBM.	Active	III	NCT00045968 (47)
GBM	CAR T cells	GD2-specific CAR T cells	GD2-specific CAR T cells activate immune responses in the tumor microenvironment.	Active	I	NCT03170141 (48)
High-grade gliomas	IFN-α	TMZ or TMZ + IFN-α	TMZ + IFN-α treatment extended the survival of patients with high-grade gliomas, especially the MGMT promoter unmethylation variant.	Active	III	NCT01765088 (49)

## 4 Discussion

Significant studies in a specific field are typically the most frequently cited, as they have made groundbreaking contributions. Bibliometric analysis provides an intuitive method to understand the advancements of a research domain, and highly cited articles are of crucial reference value for future research. Here, we are revealing an overview of the current state of immunotherapy for glioma based on top 100 most cited articles, highlighting its development trends and research hot spots, and providing guidance for future studies.

Globally, the USA has an overwhelming dominant position in this research area with most highly cited articles (n = 88). All of the top 10 most productive authors and institutions were from the USA. The USA cooperated most often with other countries/regions. Professor Heimberger AB from the University of Texas MD Anderson Cancer Center ranked first with 15 highly cited documents and received 3,086 citations. Professor Heimberger’s early research mainly focused on epidermal growth factor receptor VIII (EGFRvIII) peptide vaccine against intracerebral tumors. EGFRvIII-targeted vaccine in her studies was safe and efficacious in patients with GBM (50, 51). In recent years, Professor Heimberger has been working on the development of biomarkers for ICIs against glioma. The relationships between tumor mutation burden, mismatch repair, and immune checkpoint expression were discussed (52). Professor Sampson JH from Duke University had 12 highly cited articles and received 3,556 citations. Professors Sampson and Heimberger have collaborated closely on several studies. In addition to the study on EGFRvIII peptide vaccine, Professor Sampson also worked on developing adoptive cell therapy to treat glioma (51, 53). EGFRvIII-specific CAR T cells were efficacious in brain glioma, highlighting the significance of immunocompetent models in the investigations of glioma immunotherapy (53). In Professor Sampson’s recent study (2018), his team found that patients with untreated GBM and mice harbored low level of T cells and contracted splenic (54). T-cell dysfunction contributed to tumor immune escape in patients with cancer, which was particularly evident in GBM (54).

The top 100 most cited studies in this area were published on journals with high impact, such as Nature Medicine, Nature, Cell, and Journal of Clinical Oncology, and in other reputable journals including Clinical Cancer Research, Neuro-Oncology, Cancer Research, Science Translational Medicine, Cancer Immunology Immunotherapy, and Cancer Immunology Research. These journals can also guide groundbreaking research manuscripts for submission.

In bibliometrics, “Citation bursts” refers to a sudden increase in the number of times a particular publication is cited within a short period. The analysis of citation bursts can provide insights into the influence of a groundbreaking research publication. From **Figure 5**, the earliest reference with a citation burst was the article by Liau et al. published in 2005, and the citation burst lasted for 3 years (2008–2010) (55). Professor Liau’ research team conducted a phase I clinical trial to evaluate the efficacy and safety of dendritic cell vaccination in 25 patients with GBM eliciting systemic and local tumor T-cell responses. Their study indicated that patients with glioma with relatively small tumors, slow progression, and low TGF-beta expression were more likely to benefit from the dendritic cell vaccine (55). The most recent references with citation bursts are the articles by Brown CE, Long AH, and Reardon DA, with citation bursts lasting until 2019. Brown et al. reported that a patient with recurrent GBM had increased levels of immune cells in the cerebrospinal fluid and regression of intracranial and spinal tumors after treatment with CAR T cells targeting IL13Rα2, a clinical response that lasted for 7.5 months (25). Long et al. found that CAR tonic signaling can upregulate inhibitory receptor molecules (PD-1, Lymphocyte-activation gene 3 (LAG3), T-cell immunoglobulin and mucin-domain containing-3 (TIM-3), etc.) and mediate T-cell depletion (26). This harmful effect can be reversed by using 4-1BB instead of CD28 as the costimulatory domain (26). A growing number of studies are dedicated to CAR T-cell therapy for glioma and how to optimize this therapeutic paradigm. Reardon et al. focused on ICIs treatment for glioma (27). They found that combination treatment with anti–CTLA-4 and anti–PD-1 cured animals by increasing levels of CD8^+^ T cells and natural killer cells in the tumor microenvironment. No tumor growth was detected after intracranial tumor rechallenge in mice that responded well to the treatment, suggesting the generation of a tumor-specific immune memory response (27).

Keywords are used to understand how a field has developed. In the timeline of keywords, “macrophage ontogeny,” “microglia,” “polarization,” “mass cytometry,” “tumor mutation burden,” “sensitivity,” “msh6,” “pd-1 blockade,” and “dna repair” were the recent hot keywords (2020–2021) (**Figure 6**). From the thematic map (**Figure 7B**), we can predict that “microglia” and “polarization” as the emerging research directions should be given more consideration.

### 4.1 Limitations

This study also has some limitations. Only WoSCC database was considered. Some articles in other databases such as Scopus and Pubmed may be overlooked. Among these databases, WoSCC is the most commonly used for analyzing the highly cited literatures in a certain area. Apart from that, some recently published articles were not included with fewer citations.

## 5 Conclusion

Despite some clinical trials demonstrating responses to immunotherapy in glioma, its application in glioma still has a long way to go compared to its efficacy in hematological malignancies. Positively, novel immunotherapeutic modalities are emerging in recent years, accompanied by a profound understanding of the intricate immune landscape of glioma. Major obstacles such as the highly immunosuppressive tumor microenvironment, the antigenic heterogeneity, and low permeability of drugs will be overcome in the future.

## Data availability statement

The original contributions presented in the study are included in the article/**Supplementary Material**. Further inquiries can be directed to the corresponding author.

## Author contributions

YZ: Conceptualization, Writing – original draft. ML: Data curation, Writing – original draft. XH: Writing – original draft. ZL: Writing – original draft. YS: Investigation, Writing – review & editing. MW: Validation, Writing – original draft. TH: Methodology, Writing – review & editing. XW: Data curation, Writing – original draft. LC: Investigation, Writing – review & editing. XJ: Investigation, Writing – review & editing.
