# Supplementary material for: Emerging trends and thematic evolution of immunotherapy for glioma based on the top 100 cited articles
Source: Front Oncol. 2024 Jan 12;13:1307924. doi: 10.3389/fonc.2023.1307924 (PMC10825959; doi:10.3389/fonc.2023.1307924)
Supplement: Supplementary file 1 [file Table_1.docx]

Search strategy:

#1: TI/AB/AK= “glioma*” OR “astrocytoma” OR “glioblastoma” OR “ependymoma” OR “ganglioglioma” OR “gliosarcoma” OR “medulloblastoma” OR “oligodendroglioma” OR “GBM” OR “oligoastrocytoma” OR “glial cell tumor*”

#2 TI/AB/AK= “immunotherap*” OR “ICI” OR “ICIs” OR “CPI” OR “immune-checkpoint inhibitor*” OR “immune checkpoint inhibitor*” OR “immune-checkpoint blockade*” OR “immune checkpoint blockade*” OR “CAR-T” OR “CAR T” OR “chimeric antigen receptor T-cell*” OR “chimeric antigen receptor T cell*” OR “PD-1” OR “cytotoxic T lymphocyte-associated antigen-4” OR “CTLA-4” OR “cytokine therapy” OR “vaccine*” OR “PD-L1” OR “adoptive cell transfer therapy” OR “Adoptive cell therapy”

#1 AND #2
