# Supplementary material for: Emerging trends and thematic evolution of immunotherapy for glioma based on the top 100 cited articles
Source: Front Oncol. 2024 Jan 12;13:1307924. doi: 10.3389/fonc.2023.1307924 (PMC10825959; doi:10.3389/fonc.2023.1307924)
Supplement: Supplementary file 2 [file Table_2.docx]

The top 100 cited articles on immunotherapy for glioma

| Rank | Author | Title | Journal | Year | TC | AC/Y |
| --- | --- | --- | --- | --- | --- | --- |
| 1 | Parsa AT et al. | Loss of tumor suppressor PTEN function increases B7-H1 expression and immunoresistance in glioma | NATURE MEDICINE | 2007 | 1026 | 60.35 |
| 2 | Brown CE et al. | Regression of Glioblastoma after Chimeric Antigen Receptor T-Cell Therapy | NEW ENGLAND JOURNAL OF MEDICINE | 2016 | 958 | 119.75 |
| 3 | O'Rourke DM et al. | A single dose of peripherally infused EGFRvIII-directed CAR T cells mediates antigen loss and induces adaptive resistance in patients with recurrent glioblastoma | SCIENCE TRANSLATIONAL MEDICINE | 2017 | 893 | 127.57 |
| 4 | Keskin DB et al. | Neoantigen vaccine generates intratumoral T cell responses in phase Ib glioblastoma trial | NATURE | 2019 | 682 | 136.4 |
| 5 | Cloughesy TF et al. | Neoadjuvant anti-PD-1 immunotherapy promotes a survival benefit with intratumoral and systemic immune responses in recurrent glioblastoma | NATURE MEDICINE | 2019 | 612 | 122.4 |
| 6 | Zeng J et al. | Anti-PD-1 Blockade and Stereotactic Radiation Produce Long-Term Survival in Mice With Intracranial Gliomas | INTERNATIONAL JOURNAL OF RADIATION ONCOLOGY BIOLOGY PHYSICS | 2013 | 607 | 55.18 |
| 7 | Davis ME | Glioblastoma: Overview of Disease and Treatment | CLINICAL JOURNAL OF ONCOLOGY NURSING | 2016 | 574 | 71.75 |
| 8 | Weller M et al. | Rindopepimut with temozolomide for patients with newly diagnosed, EGFRvIII-expressing glioblastoma (ACT IV): a randomised, double-blind, international phase 3 trial | LANCET ONCOLOGY | 2017 | 561 | 80.14 |
| 9 | Sampson JH et al. | Immunologic Escape After Prolonged Progression-Free Survival With Epidermal Growth Factor Receptor Variant III Peptide Vaccination in Patients With Newly Diagnosed Glioblastoma | JOURNAL OF CLINICAL ONCOLOGY | 2010 | 554 | 39.57 |
| 10 | Bouffet E et al. | Immune Checkpoint Inhibition for Hypermutant Glioblastoma Multiforme Resulting From Germline Biallelic Mismatch Repair Deficiency | JOURNAL OF CLINICAL ONCOLOGY | 2016 | 545 | 68.13 |
| 11 | Liu BL et al. | ICP34.5 deleted herpes simplex virus with enhanced oncolytic, immune stimulating, and anti-tumour properties | GENE THERAPY | 2003 | 543 | 25.86 |
| 12 | Schumacher T et al. | A vaccine targeting mutant IDH1 induces antitumour immunity | NATURE | 2014 | 474 | 47.4 |
| 13 | Reardon DA et al. | Effect of Nivolumab vs Bevacizumab in Patients With Recurrent Glioblastoma The CheckMate 143 Phase 3 Randomized Clinical Trial | JAMA ONCOLOGY | 2020 | 468 | 117 |
| 14 | Hilf N et al. | Actively personalized vaccination trial for newly diagnosed glioblastoma | NATURE | 2019 | 454 | 90.8 |
| 15 | Hussain SF et al. | The role of human glioma-infiltrating microglia/macrophages in mediating antitumor immune responses | NEURO-ONCOLOGY | 2006 | 429 | 23.83 |
| 16 | Allen E et al. | Combined antiangiogenic and anti-PD-L1 therapy stimulates tumor immunity through HEV formation | SCIENCE TRANSLATIONAL MEDICINE | 2017 | 415 | 59.29 |
| 17 | Okada H et al. | Induction of CD8(+) T-Cell Responses Against Novel Glioma-Associated Antigen Peptides and Clinical Activity by Vaccinations With alpha-Type 1 Polarized Dendritic Cells and Polyinosinic-Polycytidylic Acid Stabilized by Lysine and Carboxymethylcellulose in Patients With Recurrent Malignant Glioma | JOURNAL OF CLINICAL ONCOLOGY | 2011 | 415 | 31.92 |
| 18 | Hegde M et al. | Tandem CAR T cells targeting HER2 and IL13R alpha 2 mitigate tumor antigen escape (Publication with Expression of Concern. See vol. 129, pg. 3464, 2019) | JOURNAL OF CLINICAL INVESTIGATION | 2016 | 407 | 50.88 |
| 19 | Desjardins A et al. | Recurrent Glioblastoma Treated with Recombinant Poliovirus | NEW ENGLAND JOURNAL OF MEDICINE | 2018 | 405 | 67.5 |
| 20 | Yu JS et al. | Vaccination with tumor lysate-pulsed dendritic cells elicits antigen-specific, cytotoxic T-cells in patients with malignant glioma | CANCER RESEARCH | 2004 | 404 | 20.2 |
| 21 | Wainwright DA et al. | Durable Therapeutic Efficacy Utilizing Combinatorial Blockade against IDO, CTLA-4, and PD-L1 in Mice with Brain Tumors | CLINICAL CANCER RESEARCH | 2014 | 400 | 40 |
| 22 | Zhao J et al. | Immune and genomic correlates of response to anti-PD-1 immunotherapy in glioblastoma | NATURE MEDICINE | 2019 | 397 | 79.4 |
| 23 | Liau LM et al. | Dendritic cell vaccination in glioblastoma patients induces systemic and intracranial T-cell responses modulated by the local central nervous system tumor microenvironment | CLINICAL CANCER RESEARCH | 2005 | 393 | 20.68 |
| 24 | Berghoff AS et al. | Programmed death ligand 1 expression and tumor-infiltrating lymphocytes in glioblastoma | NEURO-ONCOLOGY | 2015 | 381 | 42.33 |
| 25 | Nduom EK et al. | PD-L1 expression and prognostic impact in glioblastoma | NEURO-ONCOLOGY | 2016 | 354 | 44.25 |
| 26 | Schalper KA et al. | Neoadjuvant nivolumab modifies the tumor immune microenvironment in resectable glioblastoma | NATURE MEDICINE | 2019 | 328 | 65.6 |
| 27 | Lang FF et al. | Phase I Study of DNX-2401 (Delta-24-RGD) Oncolytic Adenovirus: Replication and Immunotherapeutic Effects in Recurrent Malignant Glioma | JOURNAL OF CLINICAL ONCOLOGY | 2018 | 328 | 54.67 |
| 28 | Muller S et al. | Single-cell profiling of human gliomas reveals macrophage ontogeny as a basis for regional differences in macrophage activation in the tumor microenvironment | GENOME BIOLOGY | 2017 | 322 | 46 |
| 29 | Jacob F et al. | A Patient-Derived Glioblastoma Organoid Model and Biobank Recapitulates Inter- and Intra-tumoral Heterogeneity | CELL | 2020 | 321 | 80.25 |
| 30 | Mitchell DA et al. | Tetanus toxoid and CCL3 improve dendritic cell vaccines in mice and glioblastoma patients | NATURE | 2015 | 321 | 35.67 |
| 31 | Saha D et al. | Macrophage Polarization Contributes to Glioblastoma Eradication by Combination Immunovirotherapy and Immune Checkpoint Blockade | CANCER CELL | 2017 | 314 | 44.86 |
| 32 | Johnson LA et al. | Rational development and characterization of humanized anti-EGFR variant III chimeric antigen receptor T cells for glioblastoma | SCIENCE TRANSLATIONAL MEDICINE | 2015 | 310 | 34.44 |
| 33 | Ricklefs FL et al. | Immune evasion mediated by PD-L1 on glioblastoma-derived extracellular vesicles | SCIENCE ADVANCES | 2018 | 308 | 51.33 |
| 34 | McGrail DJ et al. | High tumor mutation burden fails to predict immune checkpoint blockade response across all cancer types | ANNALS OF ONCOLOGY | 2021 | 307 | 102.33 |
| 35 | Prins RM et al. | Gene Expression Profile Correlates with T-Cell Infiltration and Relative Survival in Glioblastoma Patients Vaccinated with Dendritic Cell Immunotherapy | CLINICAL CANCER RESEARCH | 2011 | 307 | 23.62 |
| 36 | van SA et al. | Anti-LGI1 encephalitis Clinical syndrome and long-term follow-up | NEUROLOGY | 2016 | 299 | 37.38 |
| 37 | Graner MW et al. | Proteomic and immunologic analyses of brain tumor exosomes | FASEB JOURNAL | 2009 | 298 | 19.87 |
| 38 | Chongsathidkiet P et al. | Sequestration of T cells in bone marrow in the setting of glioblastoma and other intracranial tumors | NATURE MEDICINE | 2018 | 290 | 48.33 |
| 39 | Woroniecka K et al. | T-Cell Exhaustion Signatures Vary with Tumor Type and Are Severe in Glioblastoma | CLINICAL CANCER RESEARCH | 2018 | 278 | 46.33 |
| 40 | Yaghoubi SS et al. | Noninvasive detection of therapeutic cytolytic T cells with F-18-FHBG PET in a patient with glioma | NATURE CLINICAL PRACTICE ONCOLOGY | 2009 | 276 | 18.4 |
| 41 | Irani SR et al. | Faciobrachial dystonic seizures: the influence of immunotherapy on seizure control and prevention of cognitive impairment in a broadening phenotype | BRAIN | 2013 | 273 | 24.82 |
| 42 | Kim JE et al. | Combination Therapy with Anti-PD-1, Anti-TIM-3, and Focal Radiation Results in Regression of Murine Gliomas | CLINICAL CANCER RESEARCH | 2017 | 270 | 38.57 |
| 43 | Liau LM et al. | First results on survival from a large Phase 3 clinical trial of an autologous dendritic cell vaccine in newly diagnosed glioblastoma | JOURNAL OF TRANSLATIONAL MEDICINE | 2018 | 269 | 44.83 |
| 44 | Gabrusiewicz K et al. | Glioblastoma-infiltrated innate immune cells resemble M0 macrophage phenotype | JCI INSIGHT | 2016 | 269 | 33.63 |
| 45 | Curtin JF et al. | HMGB1 Mediates Endogenous TLR2 Activation and Brain Tumor Regression | PLOS MEDICINE | 2009 | 268 | 17.87 |
| 46 | Ahmed N et al. | HER2-Specific T Cells Target Primary Glioblastoma Stem Cells and Induce Regression of Autologous Experimental Tumors | CLINICAL CANCER RESEARCH | 2010 | 263 | 18.79 |
| 47 | Gatalica Z et al. | Molecular characterization of cancers with NTRK gene fusions | MODERN PATHOLOGY | 2019 | 260 | 52 |
| 48 | Omuro A et al. | Nivolumab with or without ipilimumab in patients with recurrent glioblastoma: results from exploratory phase I cohorts of CheckMate 143 | NEURO-ONCOLOGY | 2018 | 259 | 43.17 |
| 49 | Phuphanich S et al. | Phase I trial of a multi-epitope-pulsed dendritic cell vaccine for patients with newly diagnosed glioblastoma | CANCER IMMUNOLOGY IMMUNOTHERAPY | 2013 | 259 | 23.55 |
| 50 | Kmiecik J et al. | Elevated CD3(+) and CD8(+) tumor-infiltrating immune cells correlate with prolonged survival in glioblastoma patients despite integrated immunosuppressive mechanisms in the tumor microenvironment and at the systemic level | JOURNAL OF NEUROIMMUNOLOGY | 2013 | 253 | 23 |
| 51 | Majzner RG et al. | CAR T Cells Targeting B7-H3, a Pan-Cancer Antigen, Demonstrate Potent Preclinical Activity Against Pediatric Solid Tumors and Brain Tumors | CLINICAL CANCER RESEARCH | 2019 | 252 | 50.4 |
| 52 | Hodges TR et al. | Mutational burden, immune checkpoint expression, and mismatch repair in glioma: implications for immune checkpoint immunotherapy | NEURO-ONCOLOGY | 2017 | 248 | 35.43 |
| 53 | Reardon DA et al. | Glioblastoma Eradication Following Immune Checkpoint Blockade in an Orthotopic, Immunocompetent Model | CANCER IMMUNOLOGY RESEARCH | 2016 | 245 | 30.63 |
| 54 | Choi BD et al. | CAR-T cells secreting BiTEs circumvent antigen escape without detectable toxicity | NATURE BIOTECHNOLOGY | 2019 | 244 | 48.8 |
| 55 | Quek AML et al. | Autoimmune Epilepsy Clinical Characteristics and Response to Immunotherapy | ARCHIVES OF NEUROLOGY | 2012 | 244 | 20.33 |
| 56 | Di T et al. | Immunobiological Characterization of Cancer Stem Cells Isolated from Glioblastoma Patients | CLINICAL CANCER RESEARCH | 2010 | 244 | 17.43 |
| 57 | Bunse L et al. | Suppression of antitumor T cell immunity by the oncometabolite (R)-2-hydroxyglutarate | NATURE MEDICINE | 2018 | 242 | 40.33 |
| 58 | Kohanbash G et al. | Isocitrate dehydrogenase mutations suppress STAT1 and CD8(+) T cell accumulation in gliomase | JOURNAL OF CLINICAL INVESTIGATION | 2017 | 241 | 34.43 |
| 59 | Friebel E et al. | Single-Cell Mapping of Human Brain Cancer Reveals Tumor-Specific Instruction of Tissue-Invading Leukocytes | CELL | 2020 | 240 | 60 |
| 60 | Sampson JH et al. | Greater chemotherapy-induced lymphopenia enhances tumor-specific immune responses that eliminate EGFRvIII-expressing tumor cells in patients with glioblastoma | NEURO-ONCOLOGY | 2011 | 240 | 18.46 |
| 61 | Hussain SF et al. | A novel small molecule inhibitor of signal transducers and activators of transcription 3 reverses immune tolerance in malignant glioma patients | CANCER RESEARCH | 2007 | 236 | 13.88 |
| 62 | Mount CW et al. | Potent antitumor efficacy of anti-GD2 CAR T cells in H3-K27M(+) diffuse midline gliomas | NATURE MEDICINE | 2018 | 232 | 38.67 |
| 63 | Touat M et al. | Mechanisms and therapeutic implications of hypermutation in gliomas | NATURE | 2020 | 230 | 57.5 |
| 64 | Lohr J et al. | Effector T-Cell Infiltration Positively Impacts Survival of Glioblastoma Patients and Is Impaired by Tumor-Derived TGF-beta | CLINICAL CANCER RESEARCH | 2011 | 230 | 17.69 |
| 65 | Bielamowicz K et al. | Trivalent CAR T cells overcome interpatient antigenic variability in glioblastoma | NEURO-ONCOLOGY | 2018 | 229 | 38.17 |
| 66 | Samson A et al. | Intravenous delivery of oncolytic reovirus to brain tumor patients immunologically primes for subsequent checkpoint blockade | SCIENCE TRANSLATIONAL MEDICINE | 2018 | 229 | 38.17 |
| 67 | Heimberger AB et al. | Incidence and prognostic impact of FoxP3(+) regulatory T cells in human gliomas | CLINICAL CANCER RESEARCH | 2008 | 229 | 14.31 |
| 68 | Fecci PE et al. | Systemic CTLA-4 blockade ameliorates glioma-induced changes to the CD4(+) T cell compartment without affecting regulatory T-cell function | CLINICAL CANCER RESEARCH | 2007 | 229 | 13.47 |
| 69 | Han JF et al. | CAR-Engineered NK Cells Targeting Wild-Type EGFR and EGFRvIII Enhance Killing of Glioblastoma and Patient-Derived Glioblastoma Stem Cells | SCIENTIFIC REPORTS | 2015 | 227 | 25.22 |
| 70 | Nduom EK et al. | Immunosuppressive mechanisms in glioblastoma | NEURO-ONCOLOGY | 2015 | 221 | 24.56 |
| 71 | Morgan RA et al. | Recognition of Glioma Stem Cells by Genetically Modified T Cells Targeting EGFRvIII and Development of Adoptive Cell Therapy for Glioma | HUMAN GENE THERAPY | 2012 | 220 | 18.33 |
| 72 | Chang SM et al. | Perioperative complications and neurological outcomes of first and second craniotomies among patients enrolled in the Glioma Outcome Project | JOURNAL OF NEUROSURGERY | 2003 | 220 | 10.48 |
| 73 | Takenaka MC et al. | Control of tumor-associated macrophages and T cells in glioblastoma via AHR and CD39 | NATURE NEUROSCIENCE | 2019 | 217 | 43.4 |
| 74 | Dorand RD et al. | Cdk5 disruption attenuates tumor PD-L1 expression and promotes antitumor immunity | SCIENCE | 2016 | 217 | 27.13 |
| 75 | El AA et al. | An increase in CD4+CD25+FOXP3+ regulatory T cells in tumor-infiltrating lymphocytes of human glioblastoma multiforme | NEURO-ONCOLOGY | 2006 | 216 | 12 |
| 76 | Ito A et al. | Cancer immunotherapy based on intracellular hyperthermia using magnetite nanoparticles: a novel concept of heat-controlled necrosis with heat shock protein expression | CANCER IMMUNOLOGY IMMUNOTHERAPY | 2006 | 211 | 11.72 |
| 77 | Giles AJ et al. | Dexamethasone-induced immunosuppression: mechanisms and implications for immunotherapy | JOURNAL FOR IMMUNOTHERAPY OF CANCER | 2018 | 209 | 34.83 |
| 78 | Yamanaka R et al. | Vaccination of recurrent glioma patients with tumour lysate-pulsed dendritic cells elicits immune responses: results of a clinical phase I/II trial | BRITISH JOURNAL OF CANCER | 2003 | 205 | 9.76 |
| 79 | Keu KV et al. | Reporter gene imaging of targeted T cell immunotherapy in recurrent glioma | SCIENCE TRANSLATIONAL MEDICINE | 2017 | 203 | 29 |
| 80 | Johanns TM et al. | Immunogenomics of Hypermutated Glioblastoma: A Patient with Germline POLE Deficiency Treated with Checkpoint Blockade Immunotherapy | CANCER DISCOVERY | 2016 | 203 | 25.38 |
| 81 | Zhai LJ et al. | Molecular Pathways: Targeting IDO1 and Other Tryptophan Dioxygenases for Cancer Immunotherapy | CLINICAL CANCER RESEARCH | 2015 | 202 | 22.44 |
| 82 | Sampson JH et al. | EGFRvIII mCAR-Modified T-Cell Therapy Cures Mice with Established Intracerebral Glioma and Generates Host Immunity against Tumor-Antigen Loss | CLINICAL CANCER RESEARCH | 2014 | 202 | 20.2 |
| 83 | Wheeler CJ et al. | Vaccination elicits correlated immune and clinical responses in glioblastoma multiforme patients | CANCER RESEARCH | 2008 | 202 | 12.63 |
| 84 | Sampson JH et al. | An epidermal growth factor receptor variant III-targeted vaccine is safe and immunogenic in patients with glioblastoma multiforme | MOLECULAR CANCER THERAPEUTICS | 2009 | 201 | 13.4 |
| 85 | Phuong LK et al. | Use of a vaccine strain of measles virus genetically engineered to produce carcinoembryonic antigen as a novel therapeutic agent against glioblastoma multiforme | CANCER RESEARCH | 2003 | 201 | 9.57 |
| 86 | Zhang CC et al. | ErbB2/HER2-Specific NK Cells for Targeted Therapy of Glioblastoma | JNCI-JOURNAL OF THE NATIONAL CANCER INSTITUTE | 2016 | 199 | 24.88 |
| 87 | De VS et al. | Postoperative adjuvant dendritic cell-based immunotherapy in patients with relapsed glioblastoma multiforme | CLINICAL CANCER RESEARCH | 2008 | 197 | 12.31 |
| 88 | Pellegatta S et al. | Neurospheres enriched in cancer stem-like cells are highly effective in eliciting a dendritic cell-mediated immune response against malignant gliomas | CANCER RESEARCH | 2006 | 197 | 10.94 |
| 89 | Wheeler CJ et al. | Clinical responsiveness of glioblastoma multiforme to chemotherapy after vaccination | CLINICAL CANCER RESEARCH | 2004 | 196 | 9.8 |
| 90 | Iglesia MD et al. | Genomic Analysis of Immune Cell Infiltrates Across 11 Tumor Types | JNCI-JOURNAL OF THE NATIONAL CANCER INSTITUTE | 2016 | 195 | 24.38 |
| 91 | Zhang F et al. | Genetic programming of macrophages to perform anti-tumor functions using targeted mRNA nanocarriers | NATURE COMMUNICATIONS | 2019 | 192 | 38.4 |
| 92 | Gabrusiewicz K et al. | Glioblastoma stem cell-derived exosomes induce M2 macrophages and PD-L1 expression on human monocytes | ONCOIMMUNOLOGY | 2018 | 190 | 31.67 |
| 93 | Wei J et al. | RETRACTED: Glioma-Associated Cancer-Initiating Cells Induce Immunosuppression (Retracted article. See vol. 21, pg. 2189, 2015) | CLINICAL CANCER RESEARCH | 2010 | 189 | 13.5 |
| 94 | Mathios D et al. | Anti-PD-1 antitumor immunity is enhanced by local and abrogated by systemic chemotherapy in GBM | SCIENCE TRANSLATIONAL MEDICINE | 2016 | 188 | 23.5 |
| 95 | Wei J et al. | miR-124 Inhibits STAT3 Signaling to Enhance T Cell-Mediated Immune Clearance of Glioma | CANCER RESEARCH | 2013 | 187 | 17 |
| 96 | Hung AL et al. | TIGIT and PD-1 dual checkpoint blockade enhances antitumor immunity and survival in GBM | ONCOIMMUNOLOGY | 2018 | 182 | 30.33 |
| 97 | Belcaid Z et al. | Focal Radiation Therapy Combined with 4-1BB Activation and CTLA-4 Blockade Yields Long-Term Survival and a Protective Antigen-Specific Memory Response in a Murine Glioma Model | PLOS ONE | 2014 | 182 | 18.2 |
| 98 | Vik-Mo EO et al. | Therapeutic vaccination against autologous cancer stem cells with mRNA-transfected dendritic cells in patients with glioblastoma | CANCER IMMUNOLOGY IMMUNOTHERAPY | 2013 | 180 | 16.36 |
| 99 | Heimberger AB et al. | Epidermal growth factor receptor VIII peptide vaccination is efficacious against established intracerebral tumors | CLINICAL CANCER RESEARCH | 2003 | 177 | 8.43 |
| 100 | Krenciute G et al. | Transgenic Expression of IL15 Improves Antiglioma Activity of IL13R alpha 2-CAR T Cells but Results in Antigen Loss Variants | CANCER IMMUNOLOGY RESEARCH | 2017 | 176 | 25.14 |

*TC: total citations; AC/Y: average citations per year;*
